# Predictive model of neurocognitive functioning after acute coronary syndrome. A machine learning approach

**DOI:** 10.34172/jcvtr.025.33340

**Published:** 2025-09-28

**Authors:** Inês Moreira, Miguel Peixoto, Dulce Sousa, Afonso Rocha, Bruno Peixoto

**Affiliations:** ^1^Department of Social and Behavioral Sciences of University Institute of Health Sciences, Gandra, Portugal; ^2^Psychosocial Rehabilitation Laboratory, Rehabilitation Investigation Center, School of Health, Polytechnic University of Porto, Gandra, Portugal; ^3^Department of Psychology, Unidade Local de Saúde de São João, Gandra, Portugal; ^4^Cardiocare Research Group, Faculty of Medicine, University of Porto, Gandra, Portugal; ^5^Department of Physical and Rehabilitation Medicine, Unidade Local de Saúde de São João, Gandra, Portugal; ^6^Associate Laboratory i4HB - Institute for Health and Bioeconomy, University Institute of Health Sciences – CESPU, Gandra, Portugal; ^7^UCIBIO - Applied Molecular Biosciences Unit, Translational Toxicology Research Laboratory, University Institute of Health Sciences, Gandra, Portugal

**Keywords:** Cognitive impairment, HDL, Depression, Glucose, B-type natriuretic peptide, Random forest

## Abstract

**Introduction:**

The interplay between coronary disease and neurocognitive dysfunction remains unclear with several underlying factors likely contributing to this complex relationship. This study develops a predictive model using a machine learning approach to determine a predictive model of neurocognitive functioning in patients with acute coronary syndrome (ACS).

**Methods:**

Sixty-three patients, enrolled in the phase III cardiac rehabilitation program, underwent a neurocognitive assessment. To predict neurocognitive functioning a cross validated random forest model was used (RF_cv) due to its robustness to non-linear relationships and overfitting, and its successful application in prior disease prediction studies.

**Results:**

The RF_cv model showed an r-squared of 0.978, an RMSE of 0.6309 and a MAE value of 0.479. The top-ten predictors in the model were: HDL, Depression, Glucose, Glycated Hemoglobin, B-Type Natriuretic Peptide, BMI (Kg/m2), Waist-to-Hip Ratio, Cholesterol, Anxiety and Age.

**Conclusion:**

The variance in neurocognitive functioning is explained by a combination of biochemical indicators and body composition, reflecting classical cardiovascular risk factors and depression. The obtained RF-cv predictive model supports early identification of patients for tailored interventions.

## Introduction

 Cardiovascular diseases represent the main cause of morbidity and mortality in Europe and are considered major health and socioeconomic burden. ^1,2,3^ The Acute Coronary Syndromes (ACS), namely unstable angina and myocardial infarction subtypes, result from a blockage or narrowing of the coronary arteries, due to the rupture or erosion of the atherosclerotic plaque, accompanied by varying degrees of superimposed thrombosis and distal embolization, leading to sustained myocardial hypoperfusion. ^4^

 ACS are associated with an increased likelihood of developing cognitive impairment or dementia.^5^ This association was initially identified by observing that patients with previous ACS had a fivefold higher risk of developing a dementia.^6^ Furthermore, ACS has been linked to non-amnesic mild cognitive impairment, which is characterized by alterations in psychomotor skills, attention, and executive functioning. ^7^

 The prevalence of neurocognitive dysfunction after ACS varies widely, ranging from 9% to 85%.^8-10^ Deficits in executive functioning and verbal memory, decreased performance in verbal fluency tasks, psychomotor speed and mental flexibility, have been consistently reported. ^9,11,12^ ACS is associated with reduced gray matter volume in areas critical for demanding cognitive tasks, including the left medial frontal cortex, left cingulate and precuneus, and left and right parahippocampal gyri ^13^ as well as the hippocampus.^14^ In addition to structural changes, altered functional brain networks, especially those involved in higher-order cognition, have been reported.^15^ Executive dysfunction in these patients is linked to increased functional connectivity in middle-orbito-frontal regions.^16^

 The potential interplay between coronary disease and neurocognitive dysfunction remains unclear, with various underlying factors likely contributing to this complex relationship. Atherosclerotic disease, hypoperfusion and ischemia, inflammatory activation, metabolic alterations, psychosocial characteristics, and several cardiovascular risk factors such as obesity, hypertension, physical inactivity and dyslipidemia, are all elements contributing to this complex equation. ^13,17-19^ Many of these relationships are noteworthy for their non-linear nature.^19^ Given the increasing number of individuals with coronary heart disease and neurocognitive impairment, it is crucial to gain insight into pathways underlying the heart-brain connection.^5^

 Therefore, this study aims to develop a predictive model of neurocognitive functioning in patients with ACS admitted to hospital-based cardiac rehabilitation program. We have chosen a machine learning algorithm, the Random Forest (RF), due to its strong empirical performance in biomedical applications, and its robustness in small-sample settings. Additionally, when compared to linear models like logistic regression and alternatives such as Support Vector Machine and Neural Networks, RF typically offers a favorable balance of accuracy, interpretability (feature importance), and tractability with limited data.^20^

## Materials and Methods

###  Participants

 Sixty-three patients, 53 (84.1%) males, enrolled in an outpatient cardiac rehabilitation program at the Cardiac Rehabilitation Unit of the Departament of Physical Medicine and Rehabilitation at *Unidade Local de Saúde* de *São João *(Table 1). Patients with a history of neurological or psychiatric disorders, previous cardiac or cerebrovascular events, uncorrected sensory impairment, or illiteracy were excluded.

**Table 1:** Sociodemographic, Clinical, Biochemical and Functional Characteristics of Participants at Start of Cardiac Rehabilitation

	**Mean**	**SD**	**[Min.-Max.]**	**n**	**%**
Age (years)	55.06	10.3	[35-79]		
Education (years)	9.89	4.28	[2-23]		
Diagnosis					
ST-segment Elevation Myocardial Infarction				36	57.1
Non-ST-segment Elevation Myocardial Infarction				20	31.7
Unstable Angina				2	3.2
Others				5	7.9
Treatment					
Medical				12	19
Percutaneous Coronary Intervention				45	71.4
Coronary Bypass				2	3.2
Others				4	6.3
Ventricular Ejection Fraction (%)	52,31	10.32	[18-71]		
B-Type Natriuretic Peptide (BNP) (pg/ml)	102,03	97.33	[10-490.3]		
Troponin	43062,08	76137	[92.7-429468.2]		
Cholesterol (mg/dl)					
HDL	43,27	11.78	[26-85]		
LDL	114,70	43.01	[48-249]		
Triglycerides (mg/dl)	152,81	106.85	[48-692]		
Glucose (mg/dl)	102,69	24.60	[48-193]		
Glycated Hemoglobin (%)	5,89	0.87	[4.3-10.7]		
BMI (Kg/m2)	27,01	3.46	[19-36]		
Abdominal Circumference (cm)	96,76	9.21	[82-120]		
Waist-to-Hip Ratio	1,0217	0.284	[0.86-2.91]		
Presence of cardiovascular risk Factors					
Arterial Hypertension				26	41.3%
Type II Diabetes				11	17.5%
Dyslipidemia				47	74.6%
Overweight				42	66.7%
Obesity				12	19%
Physical Inactivity				37	58.7%
Smoking Habits				30	47.6%
Resting Heart Rate (bpm)	71.27	10.02	[50-104]		
Maximum Heart Rate during Exercise Test (bpm)	133.13	16.72	[76-170]		
Exercise Test Duration (min.)	8.34	2.34	[3-13.6]		
Reduction in Heart Rate after the Test (bpm)	23.42	10.03	[3-45]		

###  Neurocognitive Assessment

 All participants were assessed with the Addenbrooke’s Cognitive Examination-III (ACE-III) for neurocognitive functioning.

 The ACE-III is a screening test, enabling a wide evaluation of neurocognitive functioning. The ACE-III assesses five cognitive domains (Attention, Memory, Verbal Fluency, Language, and Visuospatial Functioning). ^21-23^ This instrument demonstrates a high level of reliability (α = 0.914) and appropriate discriminative ability for mild and major cognitive impairment.^23^ It has been already used in the context of cardiac pathology.^9,18,19^

 The obtained results were transformed into *z*-scores, according to the normalization formula based on the age and education of the participants, ^22^ to facilitate standardization and continuous modeling.

 Due to the close relationship between neurocognition and emotional functioning^24^ and the role that depression and anxiety play in the context of coronary pathology,^17^ an assessment of emotional domains was carried out using the Hospital Anxiety and Depression Scale (HADS). HADS consists of seven items to assess anxiety and seven items to assess depression. These two dimensions contribute to the total score, which corresponds to the level of emotional distress. ^25,26^

###  Applied Machine Learning Algorithms and Statistical Analysis 

 The statistical analysis was performed using *RStudio* program, version 4.3.2

 Frequencies, measures of central tendency and measured of deviation were used to characterize participants and the results obtained from the neurocognitive and emotional assessments.

 Since Random Forest (RF) models can capture non-linear relations between variables, are less sensitive to outliers and less prone to overfitting,^27-29^ a RF was performed to determine a predictive model of neurocognitive functioning. the model’s generalizability to an independent dataset, cross-validation was applied. Compared to linear models such as logistic regression, RF has consistently demonstrated superior performance in health-related datasets, making it a reliable choice when interpretability and accuracy are both important. In small-sample biomedical scenarios (e.g., pediatric HIV prognosis), RF has outperformed both linear and neural approaches in accuracy and sensitivity.^20^

 To obtain the RF model the *randomForest* and* caret *libraries were loaded.

 The *trainControl* function from the *caret* package was used to define the cross-validation control, specifying a 10-fold cross-validation. For the hyperparameters, the model was configured to use 500 decision trees, with two predictor variables randomly selected (*mtry* = 2) at each node to find the best split. The node size was set to zero, therefore allowing the algorithm to automatically choose the node size during the training of the model.

 Cross-validated predictions were generated by using the *predict* function. The model’s performance was assessed in 12 cases, not included in the model’s training, using Cross-validated R-squared, Rooted Mean Squared Error (RMSE), and Mean Absolute Error (MAE) were used.

 To prevent multicollinearity between the predictors, a cutoff value of 0.8 was used to identify and exclude highly correlated variables. Consequently, the total score of HADS was excluded due to its high correlation with both depression (*r* = .859) and anxiety (*r* = .895). Similarly, LDL was excluded for its high correlation with total cholesterol (*r* = .88). Although additional feature selection techniques (e.g., recursive feature elimination, PCA, or regularization) were not used in this study, we acknowledge their potential value and recommend them for future work.

###  Procedures

 The present research obtained approval from the Ethics Committee of *São João Hospital Center, EPE*. All participants provided informed consent.

 Study participants were recruited upon admission to the Physical Medicine and Rehabilitation service. Neurocognitive and emotional assessment was performed at the beginning of the cardiac rehabilitation program, through a trained interviewer and following standardized data collection form.

 Clinical, functional, and biochemical data and information related to cardiovascular risk profile and anthropometry were retrieved from the clinical records.

## Results

 In Table 1, clinical and biochemical data of the participants are presented, as well as the presence of cardiovascular risk factors of patients at start of CRP. The same table also depicts data on functional capacity at baseline assessment. Since the aim of this study was to predict neurocognitive functioning as a continuous outcome (z-score), and not to compare groups, no control group was included.

 In Table 2, the results obtained in the ACE-III and HADS are presented. Using a cutoff score of 8 on the HADS to identify ‘clinically significant’ emotional symptoms, 22.2% (n = 14) of the participants exhibited clinically significant levels of depressive symptoms, while 47.6% (n = 30) had clinically significant levels of anxious symptoms. The results obtained from the ACE-III indicate that 65% (n = 41) of the participants scored below 1.5 standard deviations, suggesting neurocognitive impairment.

**Table 2:** Cognitive and psycho-emotional status according to the ACE-III and HADS

	**M**	**SD**	**[Min.-Max.]**
ACE-III	-2.18	1.47	[-6.52-1.10]
HADS			
Depression	4.32	3.90	[0-16]
Anxiety	7.83	4.46	[0-17]

 The crossed-validated RF model obtained has a r-squared of 0.978, a RMSE of 0.6309 and a MAE value of 0.479, with the predictive value of each predictor depicted in Table 3. The top-ten predictors determined by the model were HDL levels, depression, glucose and glycated hemoglobin, B-Type Natriuretic Peptide levels, body mass index, waist-to-hip ratio, total cholesterol, anxiety, and age.

**Table 3:** Values of the predictors determined by the cross-validated random forest model.

	**Predictive value (importance score)**
HDL (mg/dl)	9
Depression	7.83
Glucose (mg/dl)	6.31
Glycated Hemoglobin (%)	5.80
B-Type Natriuretic Peptide (BNP) (pg/ml)	5.58
BMI (Kg/m2)	5.57
Waist-to-Hip Ratio	5.52
Cholesterol (mg/dl)	5.31
Anxiety	5.00
Age	4.86
Education (years)	4.64
Resting Heart Rate (bpm)	4.28
Packs per Year	4.13
Triglycerides (mg/dl)	3.90
Exercise Test Duration (min.)	3.62
Reduction in Heart Rate after the Test (bpm)	3.57
Ventricular Ejection Fraction (%)	2.52
Maximum Heart Rate during Exercise Test (bpm)	3.30
Troponin	3.18
Overweight	1.87
Treatment	1.4
Smoking Habits	1.24
Arterial Hypertension	0.75
Physical Activity	0.51
Dyslipidemia	0.31

## Discussion

 In this study we built, using a machine learning method, a predictive model of neurocognitive function that explained 97,86% of the variance in neurocognitive functioning, showing a good performance with low RMSE and MAE values.

 Among the top ten predictors identified by our model, HDL emerges as the most significant. Lipids play a critical role in cellular signaling, and their dysregulation has been linked to neurological disorders and the etiology of Alzheimer’s disease. ^30^, ^31^ HDL is associated with both physical and neurocognitive performance, though these relationships appear to be nonlinear and influenced by the APOE genotype.^32^ Under normal physiological conditions, HDL exhibits anti-inflammatory properties. However, during acute coronary events coupled with stress, HDL can promote inflammatory processes.^33^

 Depression is highly prevalent in patients with coronary heart disease, with major depression raging between 15 and 31.1%.^34^ It is associated with cardiovascular morbidity and mortality and significantly affects quality of life.^35^ It is internationally recognized that depression is a modifiable prognostic factor for coronary heart disease that should be systematically assessed and adequately managed. ^36^

 Depression, even outside the scope of coronary disease, is linked to a higher risk for neurocognitive decline and dementia even at mid-life^37^ accelerating brain aging.^38^

 The association between depression and coronary disease is attributed to both behavioral and biological mechanisms such as poor health behaviors, low heart-rate variability, elevated heart rate in response to stressors, elevated catecholamine levels and inflammatory activity, among others.^34^ In fact, inflammation is closely associated to depression, and there is growing evidence of its association with other cardiovascular risk factors such as cholesterol^39^ and waist circumference.^40^

 Regarding glucose and glycated hemoglobin, evidence suggests that poor glycemic control and hypoglycemia interact with vascular and emotional risk factors, linking type 2 diabetes to neurocognitive impairment. ^41,42^ Although most participants in our study did not have type 2 diabetes, the mean glycated hemoglobin value in our sample corresponds to a prediabetic stage.

 B-Type Natriuretic Peptide (BNP) is released from ventricular myocytes due to myocardial stretching and is related to the extent of infarction and left ventricular dysfunction.^43^ Elevated BNP levels have been associated to poor neurocognitive functioning in community-dwelling older adults ^44^ and with stable cardiovascular disease.^45^ In our model, BNP shows greater predictive performance than left ventricular ejection fraction, another marker of heart efficiency and neurocognitive functioning, especially important when below 50%. ^46^

 Previous studies have highlighted body mass index (BMI) and waist-to-hip ratio as key factors influencing the relationship between age and neurocognitive functioning following an ACS.^19^ Elevated midlife BMI correlates with an increased risk of dementia.^47^ Moreover, loss of lean mass and gain of fat mass, measured by waist circumference adjusted for BMI, are linked to higher risk of cerebral alterations and neurocognitive decline, primarily due to chronic inflammation.^48^

 Anxiety levels have been shown to influence the incidence of ACS, with acute stress situations leading to plaque ruptures via stress hormones, triggering an ACS.^49^ However, the neurocognitive implications of anxiety in this context are also associated with improved visuospatial and alternated attention. ^46^ This could elucidate may explain why depression exhibits higher predictive values for neurocognitive functioning compared to anxiety.

 Age and education are widely recognized factors influencing neurocognitive functioning.^22^ Neurocognitive function after ACS has been found to correlate with age, an effect moderated by BMI and waist circumference,^19^ and with education, a marker of cognitive reserve.^9^

 Previous research has aimed to determine the prevalence of neurocognitive impairments in post-ACS samples, showing a wide range of prevalences.^8,10^ Methodological differences, such as the use of different assessment tools,^10^ account for some of this variation. However, the factors identified in our study have often been overlooked and could potentially provide further insight into the variability of results. Although we used standardized ACE-III *z*-scores for modeling, this may obscure direct mapping to clinical categories such as Mild Cognitive Impairment. This trade-off between statistical modeling and diagnostic clarity warrants further exploration, possibly through parallel categorical classification models.

 The need to understand the factors mediating the relationship between ACS and neurocognitive impairment has been noted in the literature, yet our current understanding of these variables remains limited, as corroborated by other authors.^5,50^ In this context, the present study provides additional insight into the role of biochemical markers such as HDL, glucose, BNP and total cholesterol, emotional variables with a focus on depression, and constitutional aspects on neurocognitive functioning.

 The present model provides an opportunity to predict the impact of ACS on neurocognitive functioning in a clinical context, allowing early interventions such as neurocognitive stimulation to mitigate the potential impact on patients’ daily life and occupational functioning. Predictive models like the present one, could support early identification of at-risk patients in cardiac rehabilitation programs. Integration into digital health systems or clinical decision-support tools, supported by interdisciplinary collaboration, may help deliver targeted neurocognitive interventions. However, pilot studies are needed to evaluate feasibility, cost-effectiveness, and impact on patient outcomes.

 However, to test how the model generalizes to new data, it is advisable to use it in an independent sample. This will allow an assessment of the reliability and effectiveness of the model.

 Nevertheless, the complex and multifactorial relation between ACS and neurocognitive functioning calls for a multidisciplinary approach to patient care. Neurocognitive impairment and depression are often underrecognized, albeit cardiac patients with both conditions face a higher risk for major cardiovascular events.^51^ Therefore, neurocognitive and emotional assessment and intervention should be mandatory for ACS patients.

 Although machine learning practices often involve comparing multiple models, the choice of RF was made to its suitability for small datasets, resistance to overfitting, and successful record in clinical prediction tasks.^29^ Future work should include comparative analysis across different algorithms.

 Despite the strong predictive performance of the model, the relatively small sample size introduces a risk of overfitting. While 10-fold cross-validation was applied to mitigate this, such high predictive values in small datasets may not generalize well to independent populations. External validation using larger, more diverse cohorts is essential to assess the model’s robustness and real-world applicability.

 The sample’s male predominance (84.1%) is another limitation, reducing the generalizability and potentially masking sex-specific neurocognitive outcomes. We urge future research to prioritize sex-balanced cohorts to better understand gender differences in post-ACS neurocognition.

## Conclusion

 Several factors, including age, anthropometric characteristics, serum biomarkers and psycho-emotional status, can predict the risk of neurocognitive in patients surviving an ACS. The results highlight the significant role of depression in neurocognitive functioning, reinforcing the need for comprehensive neurocognitive and emotional assessments in cardiac rehabilitation programs.

 Despite the promising results, the study’s limitations, including a small sample size and male predominance, suggest the need for further validation in larger and more diverse populations. Future research should focus on validating this model in independent cohorts to enhance its generalizability and reliability.

 Implementing such predictive models in clinical practice could help identify at risk groups and facilitate early interventions, ultimately improving the quality of life and functional outcomes for ACS patients.

## Competing Interests

 None

## Ethical Approval

 Ethical approval from the Ethical Committee for Health of HSJ (CES_HSJ/FMUP_117-16).
